# Manipulation of nanoparticles of different shapes inside a scanning electron microscope

**DOI:** 10.3762/bjnano.5.13

**Published:** 2014-02-05

**Authors:** Boris Polyakov, Sergei Vlassov, Leonid M Dorogin, Jelena Butikova, Mikk Antsov, Sven Oras, Rünno Lõhmus, Ilmar Kink

**Affiliations:** 1 Institute of Solid State Physics, University of Latvia, Kengaraga 8, LV-1063, Riga, Latvia; 2 Institute of Physics, University of Tartu, Riia 142, 51014, Tartu, Estonia; 3 Estonian Nanotechnology Competence Center, Riia 142, 51014, Tartu, Estonia

**Keywords:** contact mechanics, nanomanipulation, nanoparticles, nanotribology, scanning electron microscopy (SEM)

## Abstract

In this work polyhedron-like gold and sphere-like silver nanoparticles (NPs) were manipulated on an oxidized Si substrate to study the dependence of the static friction and the contact area on the particle geometry. Measurements were performed inside a scanning electron microscope (SEM) that was equipped with a high-precision XYZ-nanomanipulator. To register the occurring forces a quartz tuning fork (QTF) with a glued sharp probe was used. Contact areas and static friction forces were calculated by using different models and compared with the experimentally measured force. The effect of NP morphology on the nanoscale friction is discussed.

## Introduction

The manipulation of nanoparticles (NPs) is a powerful method for the investigation of the mobility of nano-objects on solid substrates and it is contributing to a deeper understanding of nanomechanics and nanotribology [1]. Thanks to the rapid progress in the synthesis of NPs, there is a wide choice of materials, structures, compositions, shapes and coatings of NPs for nanomanipulation experiments. NPs demonstrate many intriguing phenomena, which are important for nanotribology and nanotechnology in general, for example low-temperature melting [2], vanishing friction [3], contact aging [4], etc.

The frictional properties of NPs have been extensively studied in manipulation experiments based on atomic force microscopy (AFM), either in contact mode or dynamic mode [5–6]. Besides the undoubted advantages of AFM manipulation methods, such as a wide choice of materials (not limited to conductive materials), and high resolution and accuracy, they have certain limitations. AFM is used for both displacement and visualization of the initial and the final position of the NPs, but these two procedures cannot be performed simultaneously. Therefore there is no real-time visual feedback in a single line scan concerning the trajectory of the particle and its type of motion (sliding, rolling or rotation) during the manipulation event. It is possible to extract trajectory and motion type data from complete AFM images as it was recently demonstrated in several works [4,7–9]. However, such a process is time consuming, since it requires a large amount of line scans to be made. The interpretation of the manipulation experiments is still a challenge because of a number of factors that might not be properly defined. Those factors include the morphology of the NP, the real contact area, as well as various diffusion and chemical processes possibly taking place in the vicinity of the NP–substrate interface [10]. For instance, in most of the nanomanipulation experiments NPs are considered to be spherical (described by the diameter only) [9,11–14], and the roughness of the substrate is neglected by assuming it to be atomically flat. Significantly fewer works are dedicated to nonspherical NPs [5,15–18] and nanoscale roughness [10].

The goal of this article is to demonstrate the advantages and to discuss the limitations of a real-time manipulation technique inside a scanning electron microscope (SEM) that is employed for tribological studies of metal NPs. The forces needed to overcome static friction and move individual polyhedron-like Au and sphere-like Ag NPs on an oxidized Si surface are measured and analyzed with respect to the morphology of the NPs.

## Experimental

The 150 nm Au NPs (BBI International) were deposited from solution onto oxidized silicon wafers (Semiconductor Wafer Inc., 50 nm of thermal oxide). The samples were annealed for 1 h at 500 °C prior to every experiment to remove the surfactant. Rounded Ag NPs were produced by laser-induced (532 nm, Expla, NL200) partial melting [19] of pentagonal Ag nanowires (Blue Nano), 120 nm in diameter, which were deposited on the oxidized silicon wafer.

High resolution images of the NPs and the traces left after displacement of the NPs were taken inside an FEI Helios Nanolab SEM. Standard contact AFM cantilevers (ATEC-CONT) were used as sharp probes to displace the NPs. The cantilever chip was mounted on a polar-coordinate manipulator Kleindiek MM3A-EM. No force registration was used inside FEI SEM.

**Figure 1:**
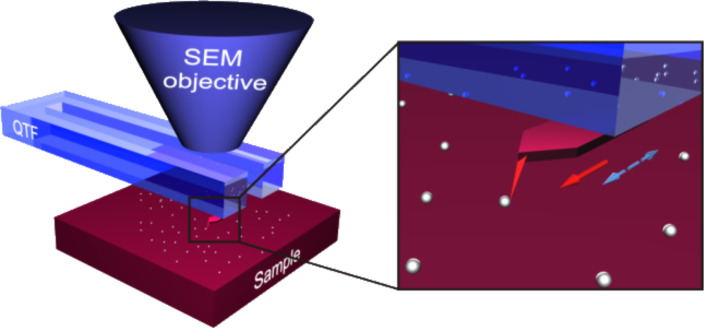
Schematics of the manipulation experiments inside an SEM. Solid arrow indicates the direction of the tip movement. Dashed arrow indicates QTF oscillation direction.

Real time manipulations with force registration were carried out inside a Vega-II SBU TESCAN SEM. The experimental set-up comprised an XYZ-nanopositioner (SLC-1720-S, SmarAct) equipped with a custom-made force sensor. The force sensor was made by gluing an electrochemically sharpened tungsten wire or commercial AFM cantilever with a sharp tip (Nanosensor ATEC-CONT cantilevers *C* = 0.2 N/m) to one prong of a commercially available quartz tuning fork (QTF). The tip of ATEC-CONT cantilevers is tilted about 15 degrees relative to the cantilever, providing visibility of the tip from the top (Figure 1). In the experiments the QTF was driven electrically on its resonance frequency in the self-excitation regime in amplitude-modulation mode. The oscillation parameters of such a system strongly depend on the load that acts on the tip, which enables one to measure the applied force. The signal from the QTF was amplified by a lock-in amplifier (SR830, Stanford Research Systems) and recorded through an ADC–DAC card (NI PCI-6036E, National Instruments). The typical values of the driving voltage were 20–50 mV and the corresponding tip oscillation amplitude was in order of 100 nm. The tip oscillated parallel to the sample surface, i.e., in the shear mode [20]. The QTF force sensors were calibrated on a reference contact mode AFM cantilever (NT-MDT, CSG11), which was previously calibrated by the thermal noise method. A detailed description of calibration procedure is given in Supporting Information File 1.

Each manipulation experiment started with a displacement of the NP from its initial position by an abrupt tip motion in the step regime to reduce the initial adhesion, which is known to be time-dependent [4]. The initial displacement was followed by a controlled manipulation of the particle by pushing it with the tip while simultaneously recording the force in the scan regime (a detailed description of SmarAct manipulator regimes is given in Supporting Information File 1). During the manipulation, the tip moved parallel to the surface along a straight line without feedback loop. At the end of every manipulation event the tip was abruptly retracted from the NP to avoid sticking of the particle to the tip. Two different modes of the tip oscillation direction were used in experiments: perpendicular to the manipulation direction and parallel to the manipulation direction.

## Results and Discussion

**Morphology of the NPs.** The shape of the NPs has a direct impact on their tribological properties and can be considered as a primary factor that determines the NP–substrate contact area and friction. The NPs used in the present experiments exhibited various morphologies, which need to be considered in more detail.

**Figure 2:**
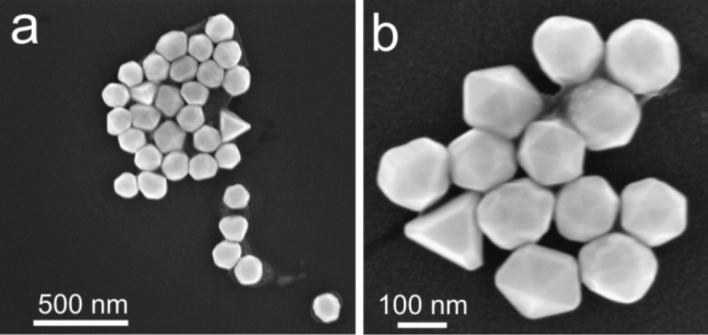
High resolution SEM images of Au NPs (150 nm) of different shape as deposited from a solution.

Intact Au NPs deposited on a substrate have well distinguishable facets (Figure 2a) and will be referred to as polyhedron-like. Tetrahedral, decahedral, icosahedral and other shapes can be identified in the SEM image (Figure 2b). Some particles exhibit truncated edges and apexes. Typical morphologies of Au NPs are listed in Table 1. It should be noted, that after the removal of the surfactant by thermal treatment partial rounding of the particles might occur [20].

**Figure 3:**
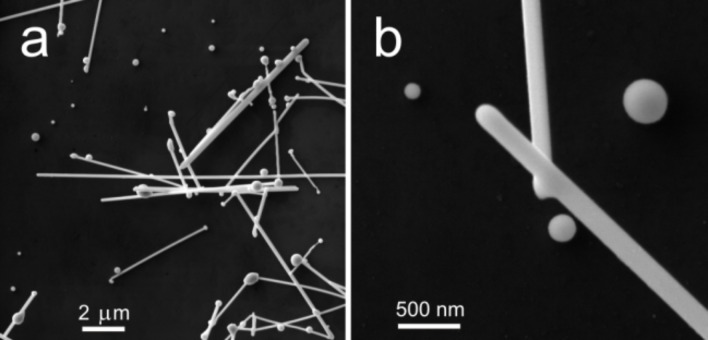
High resolution SEM images of Ag nanowires (diameter 120 nm) after pulsed laser annealing (a). Ag NPs of different size produced by laser annealing (b).

The morphology of Ag NPs was determined by the conditions of the laser annealing of the target nanowires [21–22]. At a relatively small laser pulse energy, rounded structures formed on both ends of nanowires (Figure 3a). At a higher pulse energy the whole NW melted into separated round NPs due to Plateau–Rayleigh instability [19]. All Ag NPs produced by laser induced melting appear almost spherical in the SEM micrographs (Figure 3b). There are two possible scenarios for the formation of Ag NPs. In the first scenario the molten nanostructures are able to detach from the substrate surface and solidify before contacting the substrate again [21]. In this case the particle shape can be close to ideal sphere. According to another scenario Ag nanostructures melt under the laser irradiation for a short period of time (ns) and then solidify again forming “frozen” droplets on a substrate surface. The frozen droplet model can be applied to determine the geometry of the resulted Ag NP given that the contact angle of NP–substrate interface is known. In this case the real shape of the Ag NP is a truncated sphere.

**NP–substrate contact area.** Depending on the NP morphology two basic approaches for the determination of the contact area can be distinguished: geometrical and adhesive. The contact area of polyhedron-like Au NPs on a flat substrate can be defined as the area of the facet in contact. It enables to use the geometrical approach to the contact area for faceted NPs. It should be added that because of the presence of arbitrarily truncated edges and apexes the contact areas of real particles is supposedly lower than the maximal values listed below in Table 1. For frozen-droplet shaped Ag NPs, the contact area *A* can also be estimated by geometrical considerations for a sphere of radius *R* and a cutting plane of the contact:

[1]
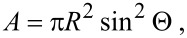


where Θ is the contact angle for Ag/SiO_2_ interface.

As described previously [20], for sphere-like NPs contact mechanics (adhesive contact approach) must be applied. The contact area is typically calculated on the basis of continuum elasticity models for deformable spheres such as the Johnson–Kendall–Roberts (JKR) [23] or the Derjaguin–Müller–Toporov (DMT-M) model [24]. According to Tabor [25], the choice of the most suitable model is determined by the parameter

[2]
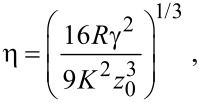


where *R* is the radius of the sphere, γ is the work of adhesion, and *z*_0_ is the equilibrium spacing for the Lennard-Jones potential of the surfaces. *K* is the combined elastic modulus of the sphere and substrate, defined as


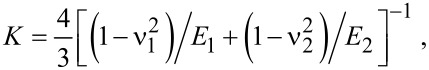


in which ν_1,2_ and *E*_1,2_ are the Poisson ratios and Young moduli of the substrate and the sphere, respectively. For small η, the DMT-M theory is more appropriate [26]. According to the DMT-M model, the contact area *A*_DMT_ of the sphere on a flat surface is:

[3]
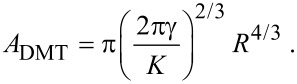


**Figure 4:**
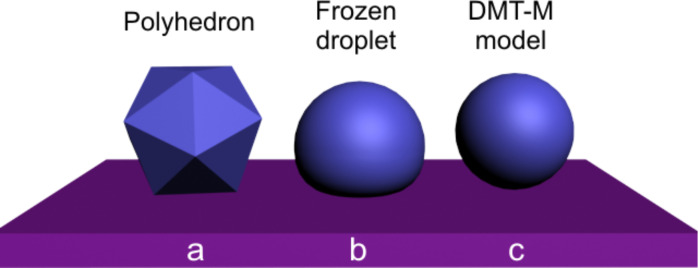
Different models for the estimation of the contact area: facet area of a polyhedron for Au NPs (a), frozen droplet for Ag NPs solidified on a substrate (b), DMT-M model for Ag NPs solidified without contact to a substrate (c).

The DMT-M approach can also be applied for the frozen droplets displaced and rolled from their initial position. For convenience different types of particle geometries and corresponding models are schematically represented in Figure 4.

**Real time manipulations of NPs.** Au and Ag NPs were manipulated inside a SEM and the lateral force, *F*_friction_, that was needed for the displacement of individual particles (i.e., static friction) was measured and analyzed with respect to the NP shape. Visual guiding during the manipulation of the NPs enabled to monitor their trajectory, in order to distinguish between continuous and abrupt motions (jumps), and to correlate the movement of the NPs with the measured tip–NP interaction force.

**Figure 5:**
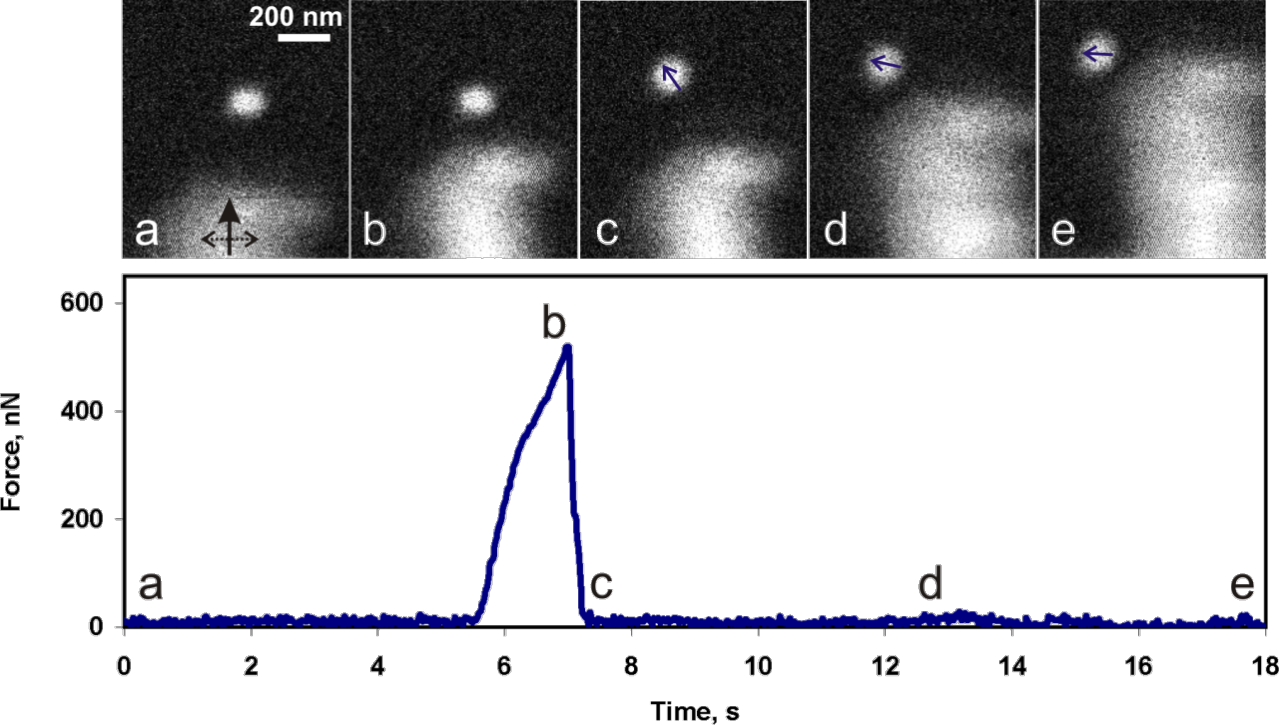
SEM snapshots of the manipulation process of a Au NP by using a tungsten tip, and the corresponding force curve. The black solid arrow in image (a) indicates the movement direction of the tip and dashed arrow shows tip the oscillation direction. The small blue arrows indicate the NP displacement directions (b, c, d).

The first series of measurements was carried out with 19 Au NPs. Figure 5 represents a typical manipulation experiment with Au NPs. The tip displacement was linear in time and the diagram presents the force curve versus time to link the SEM images and the force curve. The initial region of the curve from (a) to (b) corresponds to the tip movement at a constant height above the surface towards the NP and into the contact with the particle. The gradual increase of the force in proximity of (b) is caused by the tip–particle interaction. The peak value at point (b) corresponds to the ultimate force needed to overcome the static friction and to displace the Au particle. Usually, after overcoming the static friction the particle made a jump of a few hundred nanonewtons, and in doing so released the potential energy accumulated during loading. From (c) to (e) the particle moved smoothly in the direction that is indicated by the arrows while only a small tip–particle interaction force was exerted. The static friction in the series was found to be in the range from 50 to 750 nN. In some cases the sensor oscillation amplitude dropped to zero. This drop corresponds to a force higher than 1500–2500 nN (the upper limit depending on the particular sensor). Forces higher than these limits could not be measured due to the limited range of the QTF sensitivity at a given driving voltage.

**Figure 6:**
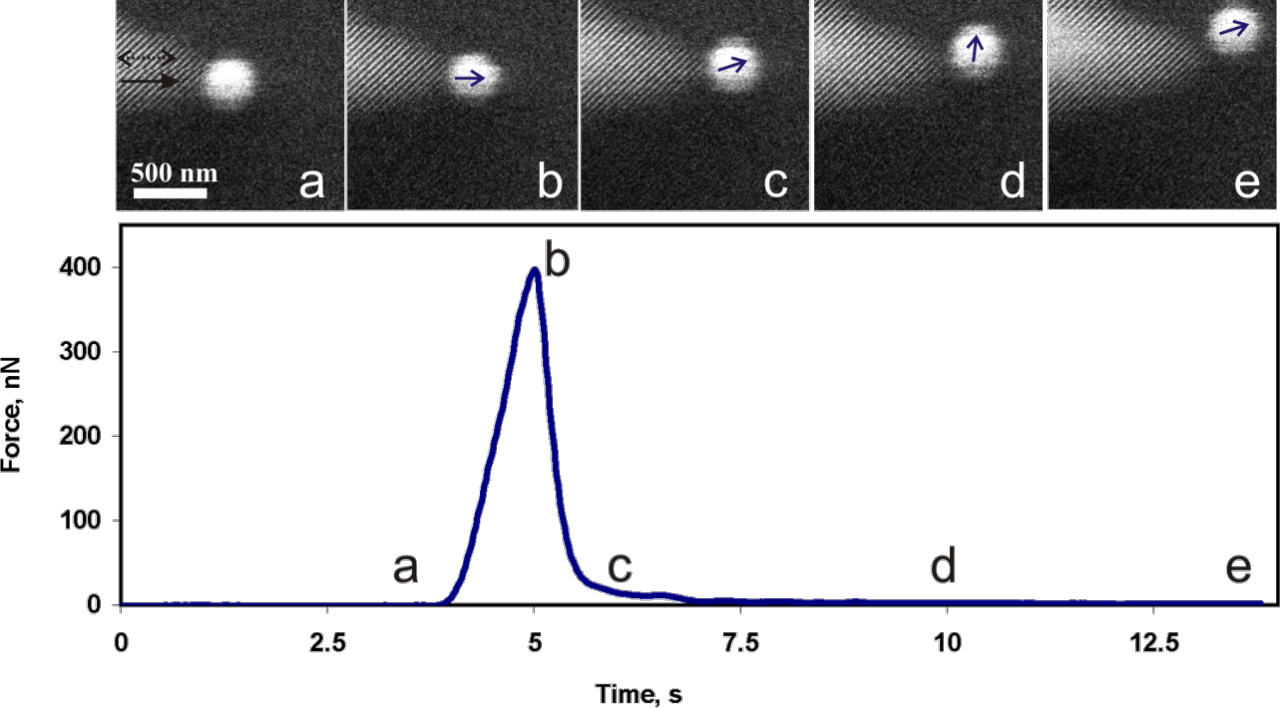
Snapshots of the manipulation of a Ag NP by using an AFM tip, and the corresponding force curve. The black solid arrow in image (a) indicates the tip movement direction and the dashed arrow shows the tip oscillation direction. The small blue arrows indicate NP displacement directions (b, c, d, e).

Another series of experiments was performed with 10 Ag NPs. In general, the behaviour of the Ag NPs during the manipulations was rather similar to that of the Au NPs. A typical manipulation force curve is shown in Figure 6. A force of several hundred nanonewtons was needed to overcome the static friction and to displace a NP (region in proximity of (b) in Figure 6), and only a few nanonewtons was enough to support the motion of the NP (regions (c)–(d)–(e), Figure 6). This finding is in agreement with those of other researchers, who have demonstrated that the kinetic friction is vanishing for clean surfaces in vacuum [3]. A nonstop motion of the NPs during the manipulations was an essential condition for staying in the vanishing friction regime, because rested NPs had a tendency to adhere strongly to the substrate. The static friction in the series was found to range from about 130 to 1880 nN.

**Figure 7:**
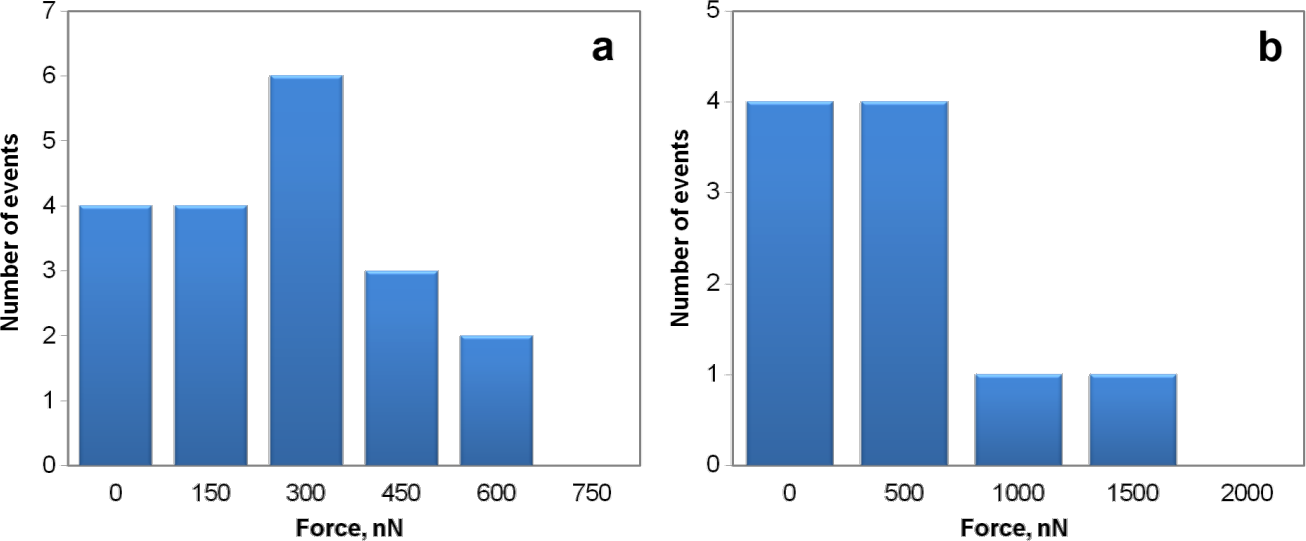
Distribution histogram of static friction force values that were experimentally measured for NPs of different shapes: polyhedron-like Au NPs (a) and for sphere-like Ag NPs (b), respectively.

The distributions of the static friction values for both polyhedron-like Au NPs and for sphere-like Ag NPs are represented in the form of histograms in Figure 7b. It should be noted that when the tip oscillated perpendicular to the manipulation direction (Figure S5, Supporting Information File 1) the NP often moved aside affected by the tip oscillation (Figure 5). Moreover, the force necessary to displace a NP can be overestimated due to an unaccounted amount of energy dissipated in a shear interaction between the tip and the NP. For the parallel tip oscillation direction (Figure S5, Supporting Information File 1) the NPs usually moved forward (motion in the manipulation direction), however, sometimes the NPs randomly deviated from the forward motion and moved aside (Figure 6). Such events are probably caused by surface defects. In summary, a tip oscillation parallel to the manipulation direction is more preferable for the manipulation of NPs because of the better control over the NP movement and a more accurate estimation of the force.

**Static friction analysis.** To analyze the results of the experimentally measured static friction force of the NPs, we calculated the friction force values, *F*_friction_, by using a simple relation:

[4]
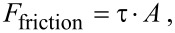


where τ is the interfacial shear stress/strength and *A* is the contact area [26]. The shear strength is defined as an ultimate shear stress τ before the object is displaced, and can be estimated by using the relation τ_theo_ = G*/*Z* between the theoretical shear strength and the combined shear modulus,


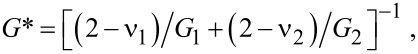


where ν is the Poisson ratio and *G*_1,2_ = *E*_1,2_/2(1 + ν_1,2_) [27–28]. *Z* is an empirical coefficient that depends on the material and ranges from 5 to 30 [29]. To calculate interfacial shear stress/strength τ for Au NPs the following parameters were used: *E*_1_ = 71.7 GPa, ν_1_ = 0.17, *E*_2_ = 78 GPa, ν_2_ = 0.36, *Z* = 30 [4]. The static friction force values for Au NPs of different geometries were calculated according to Equation 1 and presented in Table 1. The detailed calculation is given in Supporting Information File 1.

**Table 1:** Calculated contact areas and static friction forces for 150 nm Au particles of different faceting.

shape	contact area, nm^2^	static friction, nN

tetrahedral	9743	2768
decahedral	3652	1038
truncated icosahedral hexagonal facet	3474	987
icosahedral	2693	765
truncated icosahedral pentagonal facet	2301	654

Analyzing the experimentally measured and calculated static friction force values (Figure 7a and Table 1) it can be seen that the experimental friction force values mostly correspond to icosahedral and truncated icosahedral NP shapes.

**Figure 8:**
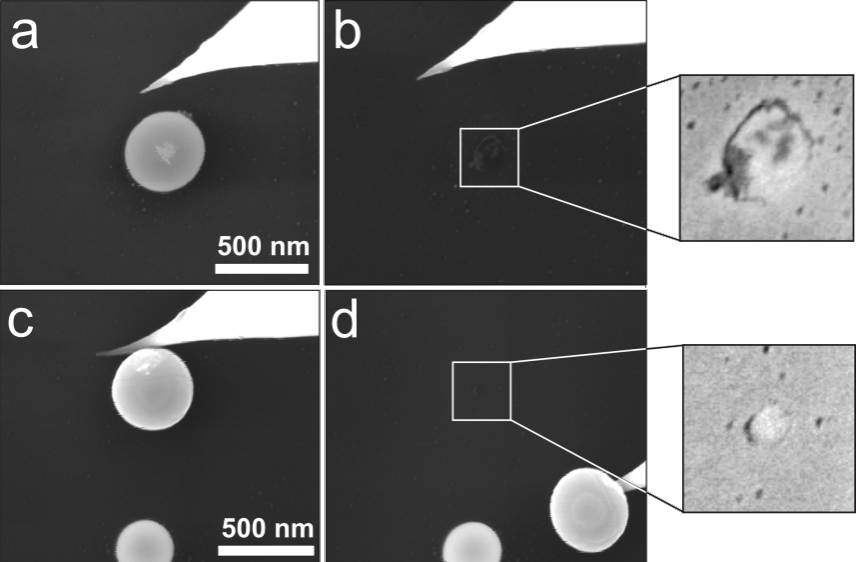
High resolution SEM images of Ag NPs (no force recording during the displacement of the NPs). Traces indicating the contact area after the first (a,b) and the second (c,d) displacement of two different Ag NPs. The corresponding estimated static friction forces are ca. 6190 nN and about 930 nN, respectively.

To evaluate the applicability of the frozen droplet and DMT-M models for Ag NPs we examined the residual traces, which remained after the displacement of 33 NPs inside a high resolution SEM. Figure 8 demonstrates the trace after the first displacement of a NP that rested on the flattened side according to the frozen droplet model (Figure 8a,b) and after the second displacement of a NP that supposedly rested on the round side according to DMT-M model (Figure 8c,d). The contact area for the first NP is ca. 21800 nm^2^, and only about 3260 nm^2^ for the second particle, which confirms our assumption that the displacement can cause a rolling of NPs from the initial flattened side to a more rounded side (and consequently decreasing the contact area).

**Figure 9:**
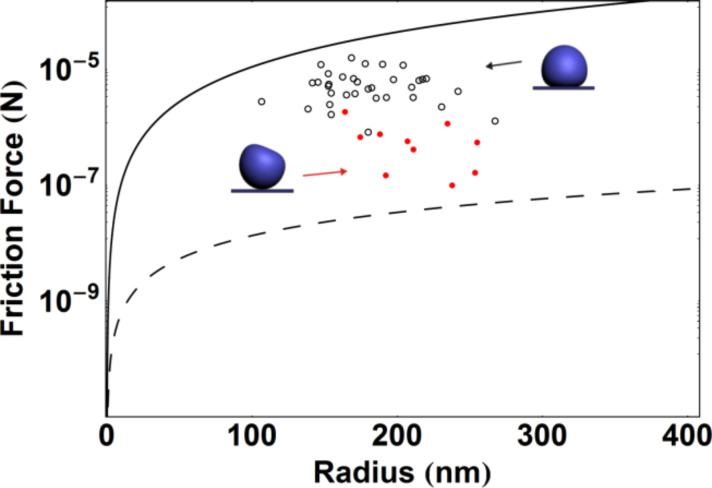
The static friction force of Ag NPs on a Si wafer as a function of the radius of the NPs. The static friction force values experimentally measured by QTF (dots) and calculated from the diameters of visible traces left after the displacement of the particles (circles). The theoretical curves of friction as functions of the radius of the NPs according to the DMT-M model (dashed curve) and frozen droplet model (solid curve).

The experimental data on the static friction forces measured by the QTF sensor (*f*_QTF_) and calculated from the diameters of the traces left after the displacement of the NPs (*f*_trace_) are shown in Figure 9 along with theoretical curves of the static friction as a function of the particle radius according to the frozen droplet (Equation 1 and Equation 4) and DMT models (Equation 3 and Equation 4). The following parameters were used in the calculations: Θ = 123.8° [30], *E*_1_ = 71.7 GPa, ν_1_ = 0.17, *E*_2_ = 82.5 GPa [31], ν_2_ = 0.36, γ = 50 mJ/m^2^ [4], and *z*_0_ = 0.3 nm, *Z* = 15 [29]. When comparing the numerical values of *f*_QTF_ and *f*_trace_ one should be aware that due to the differences between the methods only a raw estimation and qualitative conclusions can be derived. The static friction force values of NPs that were displaced for the first time (Figure 9, empty circles) are closer to the frozen droplet model. However, it is evident that the static friction predicted by the DMT-M model is smaller than the values measured with the QTF for previously displaced NPs (Figure 9, red circles). We suppose that the final shape of NPs was determined by the deviation from a perfect sphere towards an oblate spheroid according to the wettability of the liquid droplet during solidification. An alternative explanation may be the enlargement of the contact area of Ag NPs due to partial melting or enhanced diffusion of atoms caused by the electron beam (e.g., electromigration [32–33]).

**Limitations of manipulations inside a SEM.** At the end we would like to briefly discuss some limitations or drawbacks of inside-SEM nanomanipulations. The scanning rate of the electron beam is limited to a few hertz, therefore only relatively slow processes can be visualized (e.g., the monotonous motion of a NP pushed by the tip). Fast processes such as, for example, jumps of NPs can only be noticed. Another limitation is connected to the resolution of the SEM, which is related to the scanning speed and forces one to make compromises between the accuracies of determination of either the shape or the dynamics of the NPs. The effect of the electron beam on the substrate surface and on the nanostructures should also be taken into account. As it is well known, the melting temperature of the nanostructures decreases when decreasing the diameters of the structures [34–35]. Potentially, a focused e-beam is capable of introducing significant amounts of energy and causing a partial melting of the nanostructures. Additional effects can be an activation of the substrate surface or an electrostatic charging, which can also influence the results of nanotribological experiments [36].

## Conclusion

Polyhedron-like Au and sphere-like Ag NPs were manipulated on an oxidized Si substrate inside a SEM with a simultaneous force detection by using a QTF-based sensor. Real time manipulations benefit from a visual guidance of the SEM. This enables revealing the NP trajectories, distinguishing continuous or jumping motions and correlating them with the interaction force between tip and NPs. The contact areas were calculated from geometrical considerations for polyhedron-like NPs. For sphere-like NPs the contact areas were calculated by using DMT-M and frozen droplet models. The recorded static friction force values are in good agreement with the calculated values for both polyhedron-like and sphere-like NPs.

## Supporting Information

The Supporting Information contains details of SmarAct manipulator working regimes, the QTF force sensor calibration and calculations of the surface areas for different geometries.

File 1 Additional experimental details.
