# Supplementary material for: Manipulation of nanoparticles of different shapes inside a scanning electron microscope
Source: Beilstein J Nanotechnol. 2014 Feb 5;5:133–40. doi: 10.3762/bjnano.5.13 (PMC3943919; doi:10.3762/bjnano.5.13)

Supporting Information

for

Manipulation of nanoparticles of different shapes inside a scanning electron microscope

Boris Polyakov[1], Sergei Vlassov*[1,2,3], Leonid M. Dorogin[2,3], Jelena Butikova[1], Mikk Antsov[2,3], Sven Oras[2,3], Rünno Lõhmus[2,3] and Ilmar Kink[2,3]

Address: [1]Institute of Solid State Physics, University of Latvia, 8 Kengaraga Street, Riga, LV-1063, Latvia; [2]Institute of Physics, University of Tartu, Riia 142, 51014, Tartu, Estonia and [3]Estonian Nanotechnology Competence Center, Riia 142, 51014, Tartu, Estonia

Email: Sergei Vlassov* - vlassovs@ut.ee

* Corresponding author

Additional experimental details

Scan and step regimes of the nanomanipulator

The SmarAct XYZ-nanomanipulator enables two types of movement: In the single-line scan regime (hereinafter the ‘‘**scan regime**’’), the movements are made by expansion or contraction of the piezo-nanomanipulator to which the sensor is fastened. This regime provides an atomically smooth and accurate motion. In **step regime**, movements are made in a series of gradual expansions of the piezo-nanomanipulator followed by abrupt slips achieved via a sawtooth signal sent to the nanomanipulator (Figure S1). More information can be found at http://www.smaract.de/

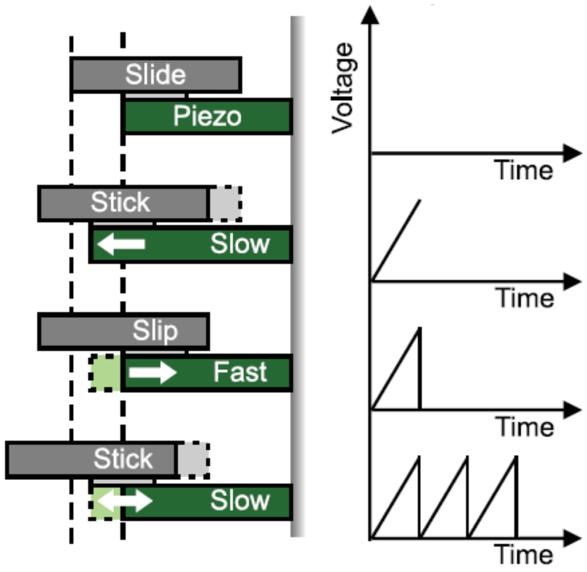

Figure S1: Step regime of the SmarAct nanomanipulator.

QTF force sensors calibration procedure

The QTF force sensors were calibrated on a reference contact mode AFM cantilever (force constant c = 0.056 Nm), which was previously calibrated by the thermal noise method. The QTF sensor mounted on the nanomanipulator was pushed against the reference cantilever by using the continuous step regime of the manipulator as shown in Figure S2. The force exerted by the reference cantilever on the QTF sensor was calculated by multiplication of cantilever displacement and cantilever force constant. QTF amplitude and x-axis displacement signal from the manipulator were recorded simultaneously (Figure S3). However, for conversion of QTF amplitude into force units, the force should be plotted as a function of QTF amplitude (Figure S4), and a fit should be found for $Force = f(\mathrm{Amplitude}_{\mathrm{QTF}})$. The calibration procedure

was similar for both sensors oscillating in parallel or in the direction perpendicular to the manipulation direction (Figure S5).

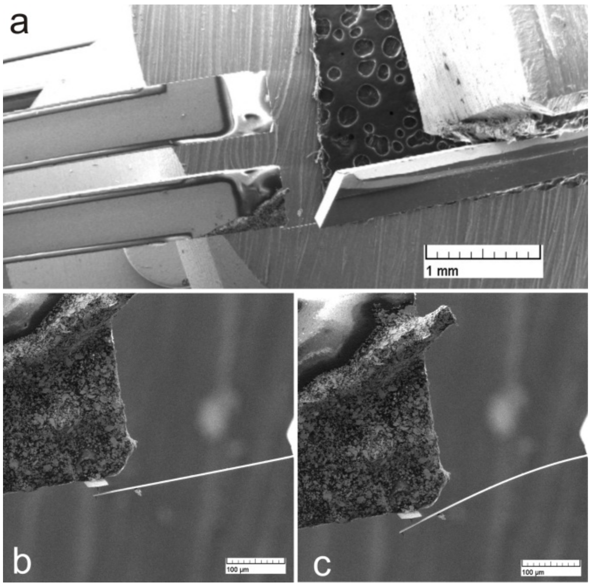

Figure S2: Calibration of the QTF sensor of reference AFM cantilever

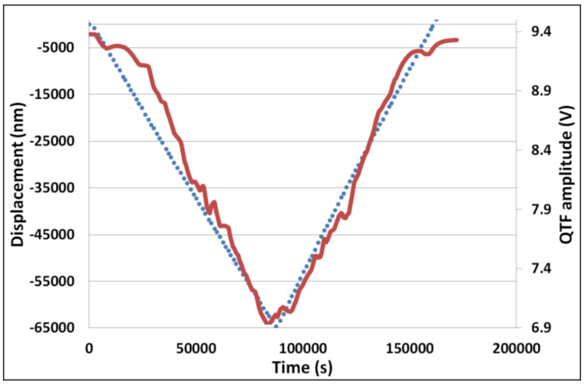

Figure S3: QTF amplitude (solid red line) and x-axis displacement signal from the manipulator (dotted line) recorded simultaneously.

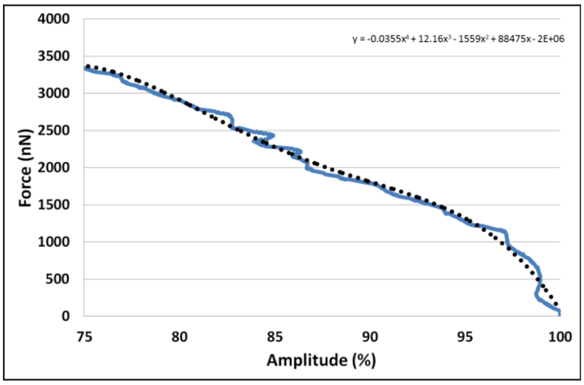

Figure S4: Force as a function of the QTF amplitude obtained during the calibration procedure. Experimental curve (solid blue line) and fitted curve (dotted line).

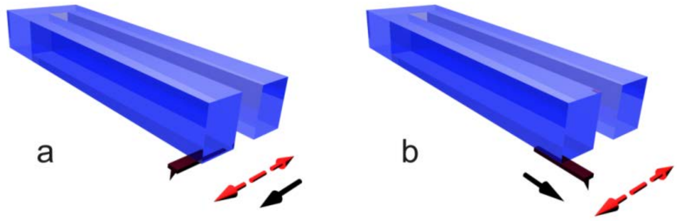

Figure S5: Oscillation directions of the sensor. (a) Parallel and (b) perpendicular to the manipulation direction.

Calculation of surface areas for different geometries

Tetrahedron

The diameter, *d*, is the height of the tetrahedron.

$$A = \frac{3\sqrt{3}d^2}{8}$$

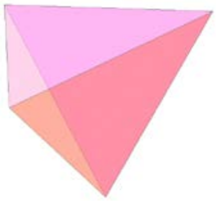

Pentagonal dipyramid

The diameter, *d*, is taken from the cross-section of the pyramid. It is the length from a tip of the pentagon to the middle of the opposite edge

$$A = \frac{\sqrt{3}d^2}{4(\cos(18°) + \cos(54°)}$$

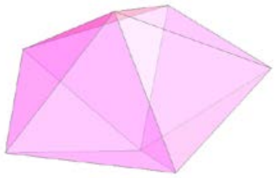

Icosahedron

The diameter, *d*, is the length between two opposite vertices.

$$A = \frac{8\sqrt{3}d^2}{4(13 + 4\sqrt{5})}$$

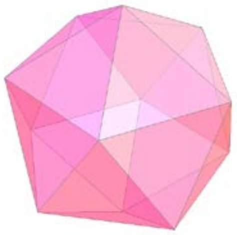

Truncated icosahedron

The diameter, *d*, is the length between two pentagonal facets.

$$A_p = \frac{d^2\sqrt{\frac{2\sqrt{5}}{5}+1}}{\frac{41\sqrt{5}}{10}+\frac{25}{2}}$$

$$A_h = \frac{3\sqrt{3}d^2}{2(\frac{41\sqrt{5}}{10}+\frac{25}{5})}$$

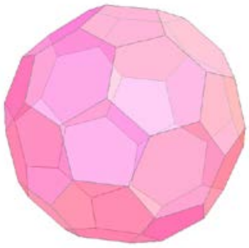

Sphere on a plane (DMT-M)

$$A = \pi(\frac{2\pi\gamma}{K})^{\frac{2}{3}}R^{\frac{4}{3}}$$

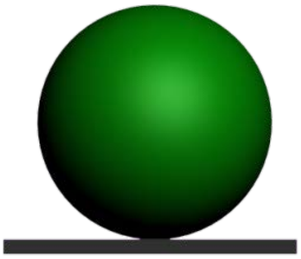

Sphere cut by a plane (frozen droplet model)

Cross-section view of a sphere cut by a plane at the contact angle Θ.

$A = \pi r^2 = \pi R^2 \sin^2 \Theta$, where r is the radius of the circle formed at the cutting interface and R is the radius of the sphere.

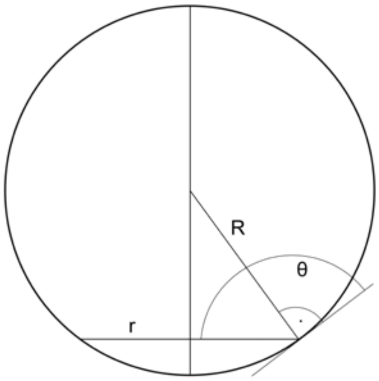

Supplement: File 1 — Additional experimental details. [file Beilstein_J_Nanotechnol-05-133-s001.pdf]
